# Hidden in Hydronephrosis: Mixed Epithelial and Stromal Tumor of the Kidney (MESTK) Mimicking Hydronephrosis

**DOI:** 10.7759/cureus.103874

**Published:** 2026-02-18

**Authors:** Simha Swaraj Sirivela, Owais Ahmed Patel, Rohan Rajendran, Giridhar P Nair

**Affiliations:** 1 Urology, Amrita Institute of Medical Sciences, Kochi, IND

**Keywords:** cystic renal lesion, er/pr positive, hydronephrosis, mestk, nephrectomy

## Abstract

Mixed epithelial and stromal tumor of the kidney is an uncommon renal lesion characterized by cystic architecture and hormonally responsive stromal components, which explains its predominance in women, particularly those with prolonged or fluctuating estrogen exposure. Its occurrence in men is unusual and can make diagnosis challenging. We present a case of a 43-year-old male who presented with vague abdominal discomfort and was found to have a markedly enlarged, non-functioning right kidney on imaging. Initial investigations suggested obstruction of the distal ureter and a non-functioning right kidney. The patient underwent laparoscopic nephrectomy, and histopathological evaluation of the tissue demonstrated features compatible with mixed epithelial and stromal tumor of the kidney (MESTK). The patient recovered uneventfully and remained well at the six-month follow-up. This case demonstrates how the tumor can present with an extreme cystic pattern that mimics chronic obstructive pathology, leading to a radiologic impression that differs from the underlying pathology. Recognizing such atypical imaging appearances may assist clinicians in considering this entity when evaluating complex cystic renal lesions, particularly in situations where clinical and radiologic findings do not align.

## Introduction

Mixed epithelial and stromal tumor of the kidney (MESTK) is a rare and usually benign renal neoplasm composed of both adenomatous epithelial elements and spindle-cell stromal components. The tumor was first described by Michal and Syrucek in 1998 [1]. These tumors contain ovarian-like stroma and typically show estrogen and progesterone receptor positivity on immunohistochemical staining, as further defined in subsequent studies [2,3].

Most MESTK lesions are seen in women, suggesting a hormonal influence in their pathogenesis [3]. Due to their rarity in men, diagnosis is often challenging, particularly when the clinical picture mimics more common conditions like renal malignancy or obstructive uropathy. We present a case of MESTK in a middle-aged male in which the lesion radiologically resembled a non-functioning hydronephrotic kidney.

## Case presentation

**Figure 1:**
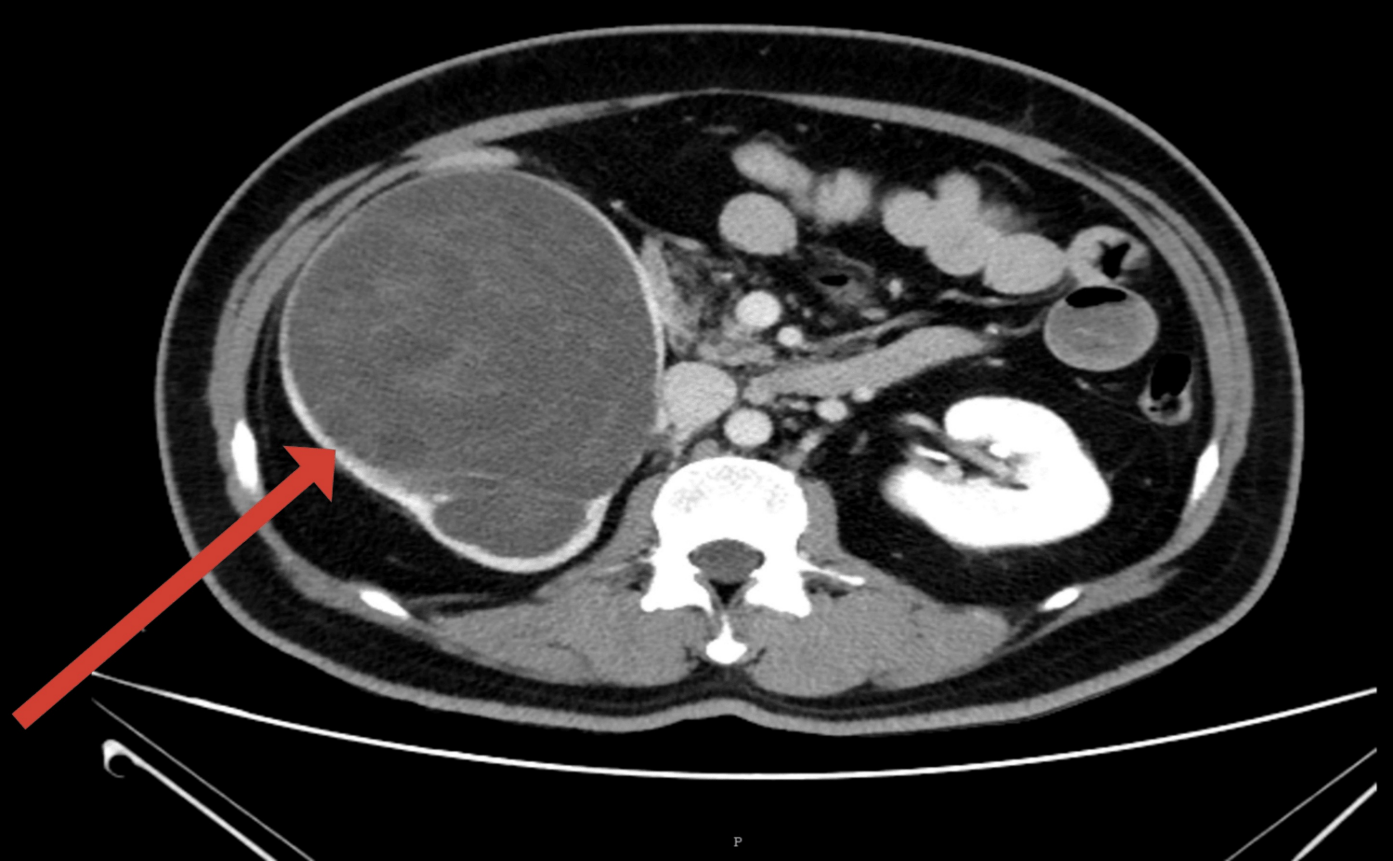
Axial contrast-enhanced CT image showing gross right hydroureteronephrosis with thinned-out renal parenchyma and no visible mass or septations. The arrow points to the right kidney, which is enlarged and appears cystic, without septations (radiologically mimicking hydronephrosis).

**Figure 2:**
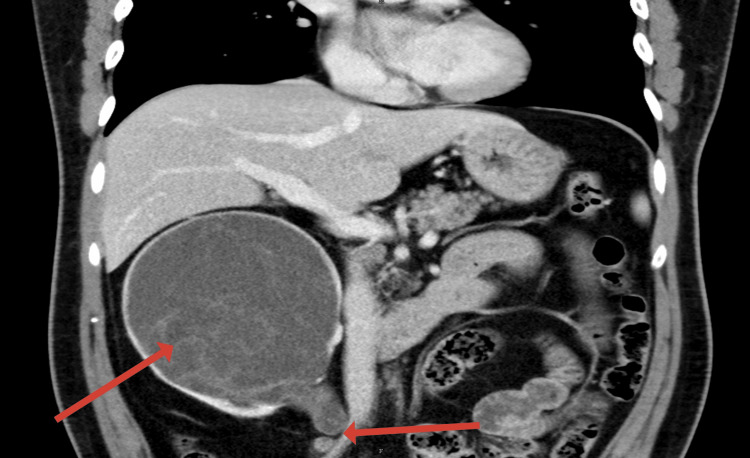
Coronal CT image demonstrating the abrupt tapering of the right proximal ureter at the iliac crossing, suggestive of distal ureteric stricture. The first arrow points to the enlarged right kidney, which has a cystic appearance, and the second arrow points to tapering of the ureteric segment, which was suspected to represent a stricture.

**Figure 3:**
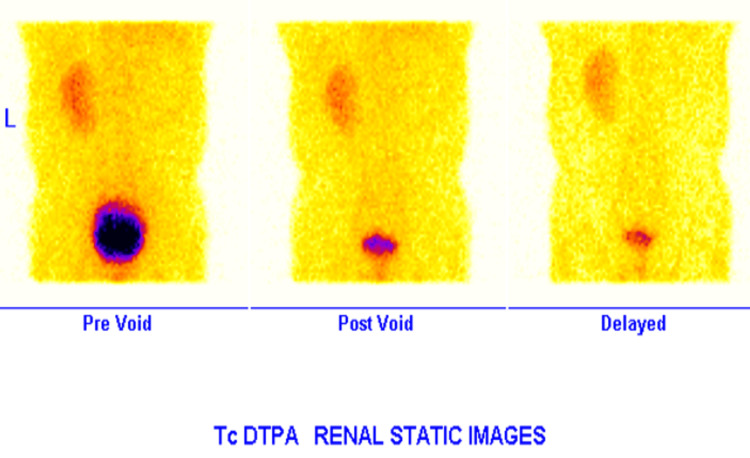
Tc-99m DTPA renogram showing normal left renal function with a non-functioning right kidney. DTPA: diethylenetriaminepentaacetic acid

A 43-year-old male presented in October of 2024 with early satiety and vague upper abdominal discomfort persisting for three months. He had no urinary complaints, fever, weight loss, or hematuria. Physical examination was unremarkable, and routine blood investigations were within normal limits. Ultrasonography revealed a large cystic lesion in the right renal fossa. Contrast-enhanced CT showed gross hydroureteronephrosis with cortical thinning and demonstrated abrupt tapering of the proximal ureter without an identifiable enhancing intraluminal lesion (Figures 1, 2). In the absence of septations, mural nodularity, or solid enhancing components within the cystic renal unit, these findings were interpreted as suggestive of a distal ureteric stricture at the level of the iliac vessels leading to chronic obstructive uropathy. Tc-99m diethylenetriaminepentaacetic acid (DTPA) renogram confirmed a non-functioning right kidney (Figure 3). Given the non-salvageable functional status of the renal unit, additional diagnostic evaluations, such as retrograde pyelography, ureteroscopy, or MR urography, were not pursued, as they were unlikely to alter definitive management.

A provisional diagnosis of obstructive uropathy with loss of renal function was made, and laparoscopic right nephrectomy was planned. Intraoperatively, the kidney was grossly enlarged, and approximately 600 mL of clear fluid was aspirated. The renal capsule was thinned and tense, and the upper ureter appeared thickened and fibrotic. The specimen was removed en bloc and sent for histopathological examination.

Gross pathology revealed a 19×17×6 cm kidney with the renal parenchyma entirely replaced by multiloculated cysts. No solid components or hemorrhagic or necrotic areas were identified. The cysts contained clear serous fluid. The attached ureter measured 15 cm and appeared diffusely thickened.

**Figure 4:**
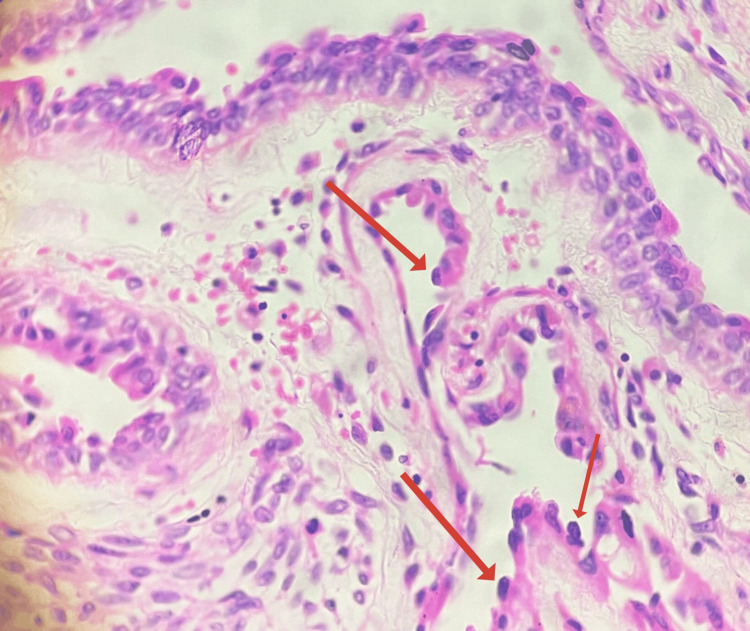
Histopathological image (H&E stain, 40x) showing cysts lined by hobnailed epithelium with surrounding ovarian-type spindle-cell stroma (arrows pointing towards hobnailed epithelium).

Microscopically, the cysts were lined by flattened to hobnail cuboidal epithelium with mild nuclear pleomorphism and hyperchromasia. The intervening stroma consisted of plump spindle cells with vesicular nuclei in a collagenous background (Figure 4). No atypia or mitotic figures were observed. Extension of the lesion into the ureteric wall was demonstrated, suggesting that the ureteric narrowing observed radiologically was intrinsic in nature due to tumor involvement rather than secondary extrinsic compression or fibrosis. There was no involvement of vascular or hilar structures. Immunohistochemistry demonstrated strong nuclear positivity for progesterone receptors (PR) and weak positivity for estrogen receptors (ER) in the stromal cells. These findings were diagnostic of MESTK.

The patient had an uneventful postoperative recovery and was discharged on the third postoperative day. At six months of follow-up, he remains asymptomatic with no evidence of disease recurrence. In view of the rare but reported potential for malignant transformation, he has been advised long-term postoperative surveillance consisting of periodic clinical assessment and annual imaging follow-up for at least five years following surgical resection.

## Discussion

Mixed epithelial and stromal tumor of the kidney (MESTK) is an uncommon biphasic renal neoplasm composed of epithelial elements and hormonally responsive stroma, as described in early characterizations [1,2]. While most cases occur in peri-menopausal women, likely related to estrogen and progesterone receptor activity within the stromal component, an increasing number of reports document occurrences in men [2,4]. These male cases, though rare, are well recognized and may present with atypical radiologic features, making preoperative diagnosis challenging [4,5]. The present case contributes to this subset by demonstrating MESTK in a 43-year-old man with no history suggestive of hormonal influence.

Radiologically, MESTK typically presents as a multiloculated cystic mass without significant solid components and is often classified within Bosniak II-III categories [3,5]. However, atypical variants have been reported that mimic hydronephrosis or benign obstructive pathology. Chou et al. described cases misinterpreted as chronic obstruction due to extensive cystic replacement of renal parenchyma [6]. In our patient, imaging showed a grossly hydronephrotic, non-functioning kidney without discernible mural nodules, closely paralleling these atypical presentations and explaining the initial impression of distal ureteric stricture. The differential diagnosis for such radiological findings includes chronic hydronephrosis secondary to stricture or calculi, multicystic dysplastic kidney, cystic nephroma, and multilocular cystic renal cell carcinoma [2,3].

Histologically, MESTK is characterized by cysts lined with flattened to hobnailed epithelium and stroma resembling ovarian tissue, which demonstrates estrogen and progesterone receptor positivity. These features, well established in the literature, were present in our specimen [2,4]. Estrogen receptors/progesterone receptors (ER/PR) positivity in male patients has been reported, supporting the concept that stromal differentiation reflects intrinsic tumor biology rather than systemic hormonal influences [4,7].

Although MESTK is typically benign, malignant transformation, including sarcomatoid progression or high-grade epithelial malignancy, has been documented. Similar behavior was noted in case series by Zou et al. and Chou et al., as well as in studies by Caliò et al., which support long-term surveillance following resection [5-7].

Surgical excision remains the standard of care for both diagnosis and treatment. Complete removal has been shown to produce excellent outcomes and mitigate the uncertainty associated with atypical imaging findings [3,4].

In summary, this case illustrates that MESTK can occur in men and may radiologically mimic hydronephrosis, complicating preoperative diagnosis. When cystic renal lesions present with discordant clinical and imaging findings, MESTK should be considered. Definitive diagnosis relies on histopathology, and surgical excision offers an excellent prognosis, with follow-up recommended due to the rare possibility of malignant transformation.

## Conclusions

Mixed epithelial and stromal tumor of the kidney (MESTK), although rare, should be considered in the differential diagnosis of complex or atypical cystic renal lesions, particularly when radiological and histological findings are discordant. This case highlights the diagnostic limitations of preoperative imaging in extreme cystic variants of MESTK, particularly when presenting as a hydronephrosis-like lesion without typical radiologic features of a cystic renal neoplasm. Recognition of this atypical presentation is essential, as definitive diagnosis may only be established following histopathological evaluation after surgical excision, with long-term follow-up recommended due to the rare possibility of malignant transformation.
